# Supplementary material for: Homogeneity of lithium distribution in cylinder-type Li-ion batteries
Source: Sci Rep. 2015 Dec 18;5:18380. doi: 10.1038/srep18380 (PMC4683395; doi:10.1038/srep18380)
Supplement: Supplementary Information [file srep18380-s1.pdf]

Supplementary Information

Homogeneity of lithium distribution in cylinder-type Li-ion batteries

A. Senyshyn[1,*] , M.J. Mühlbauer[1,2], O. Dolotko[1], M. Hofmann[1], H. Ehrenberg[2,3]

[1]*Heinz Maier-Leibnitz Zentrum (MLZ), Technische Universität München, Lichtenbergstr. 1, 85748 Garching, Germany*

[2]*Institute for Applied Materials (IAM), Karlsruhe Institute of Technology (KIT), Hermann-von-Helmholtz-Platz 1, D-76344 Eggenstein-Leopoldshafen, Germany*

[3]*Helmholtz-Institute Ulm for Electrochemical Energy Storage (HIU), P.O. Box 3640, D-76021 Karlsruhe, Germany*

*Correspondence and requests for materials should be addressed to A. S. (anatoliy.senyshyn@gmail.com)

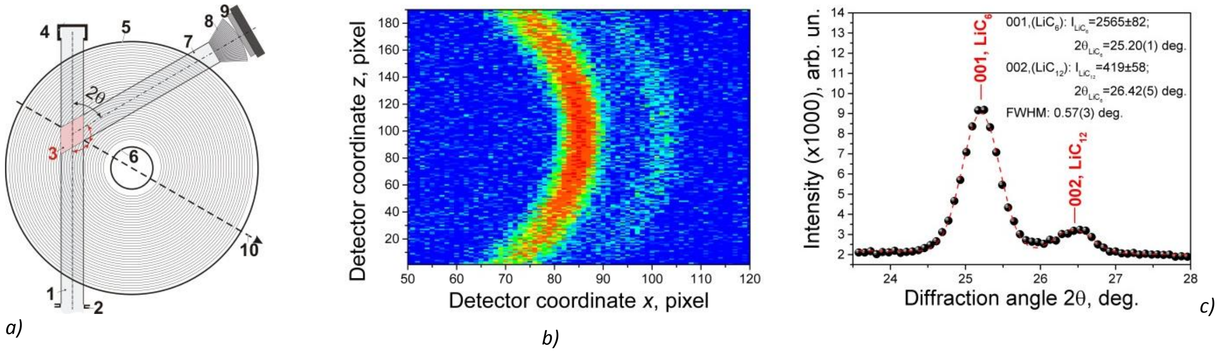

Fig. S1. (a) Sketch of experimental setup, 1 and 7 correspond to the incident and elastically scattered neutron beam, 3 is the effective gauge volume (marked by red) as formed by a system of primary slits 2 and a radial oscillating collimator 8; 4 is the direct beam catcher, 5 and 6 are the cell housing and centre pin, respectively; 9 is the 2D neutron detector and 10 indicates the scanning (bisecting) direction x. (b) Typical diffraction image obtained by the 2D neutron detector and (c) the diffraction pattern obtained by integration over the detector height with two Gaussians fitted to it.

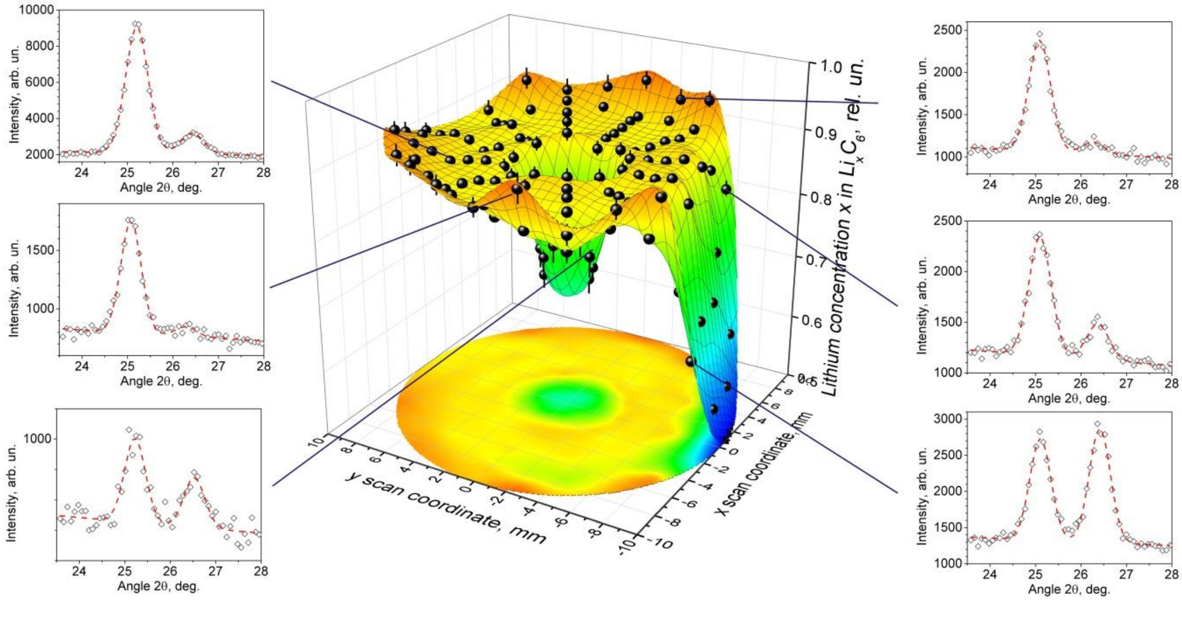

Fig. S2. Spatial distribution of mean lithium concentration in cell 1. Experimental data are shown by black points and surfaces in false color representation (similar to ones the used in Figs. 2) display the interpolation results. Insets illustrate obtained diffraction data at selected coordinates.

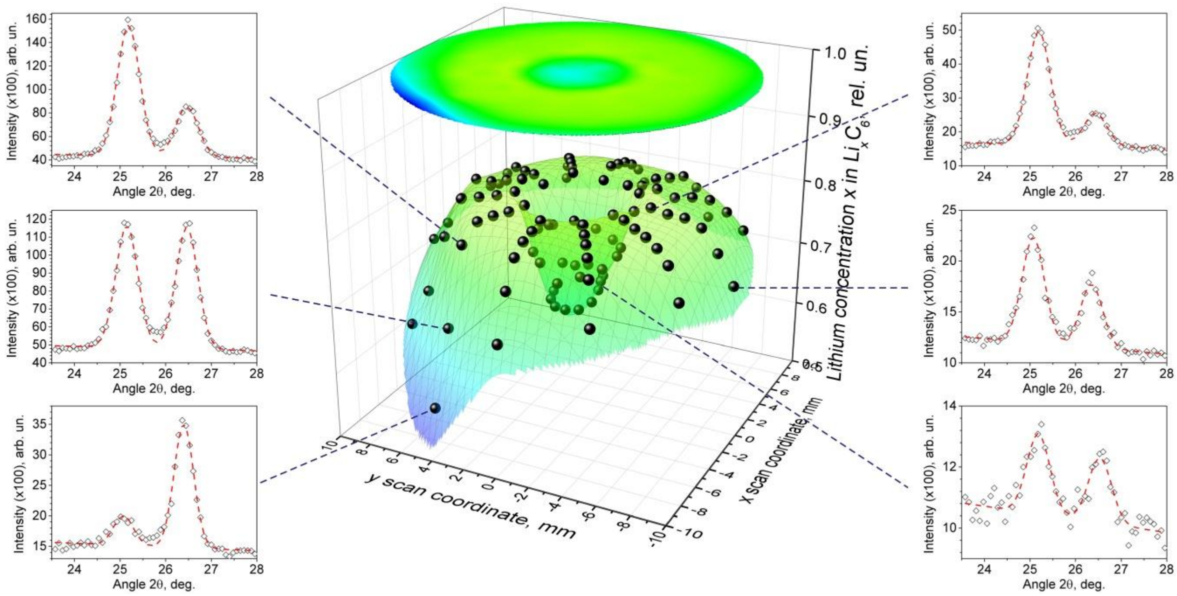

Fig. S3. Spatial distribution of mean lithium concentration in cell 2. Experimental data are shown by black points and surfaces in false color representation (similar to ones the used in Figs. 2) display the interpolation results. Insets illustrate obtained diffraction data at selected coordinates.

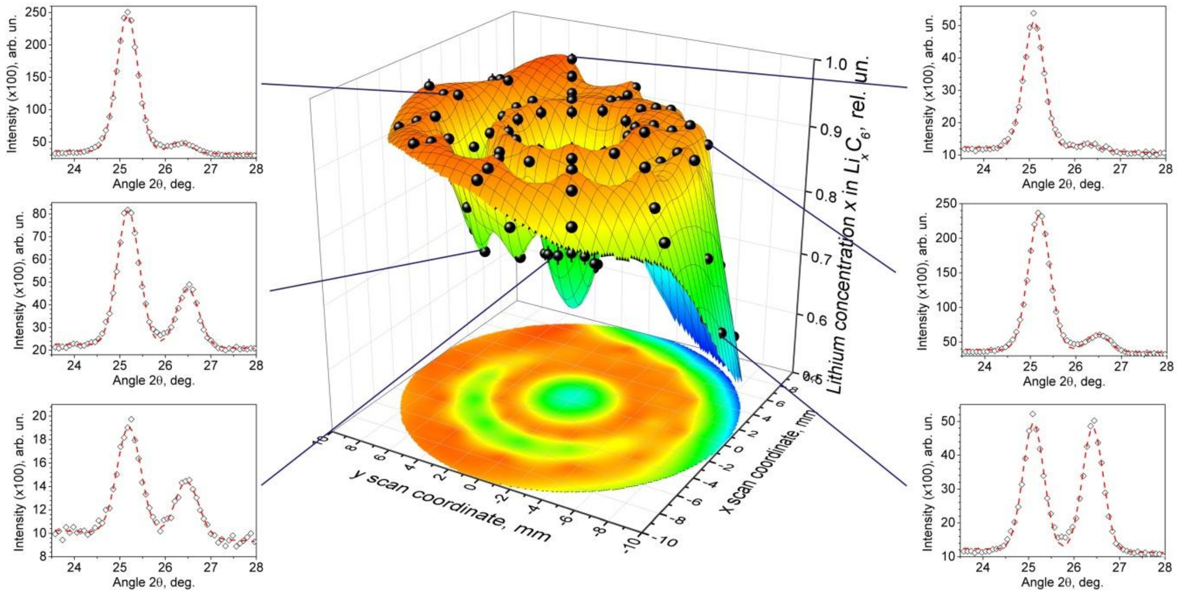

Fig. S4. Spatial distribution of mean lithium concentration in cell 3. Experimental data are shown by black points and surfaces in false color representation (similar to ones the used in Figs. 2) display the interpolation results. Insets illustrate obtained diffraction data at selected coordinates.

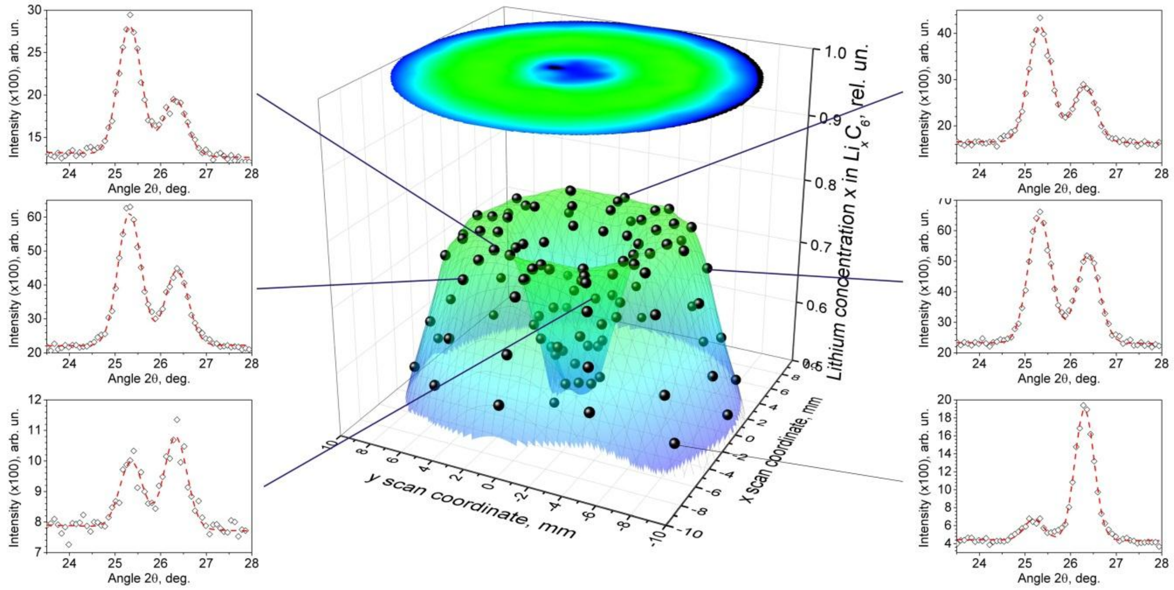

Fig. S5. Spatial distribution of mean lithium concentration in cell 4. Experimental data are shown by black points and surfaces in false color representation (similar to ones the used in Figs. 2) display the interpolation results. Insets illustrate obtained diffraction data at selected coordinates.

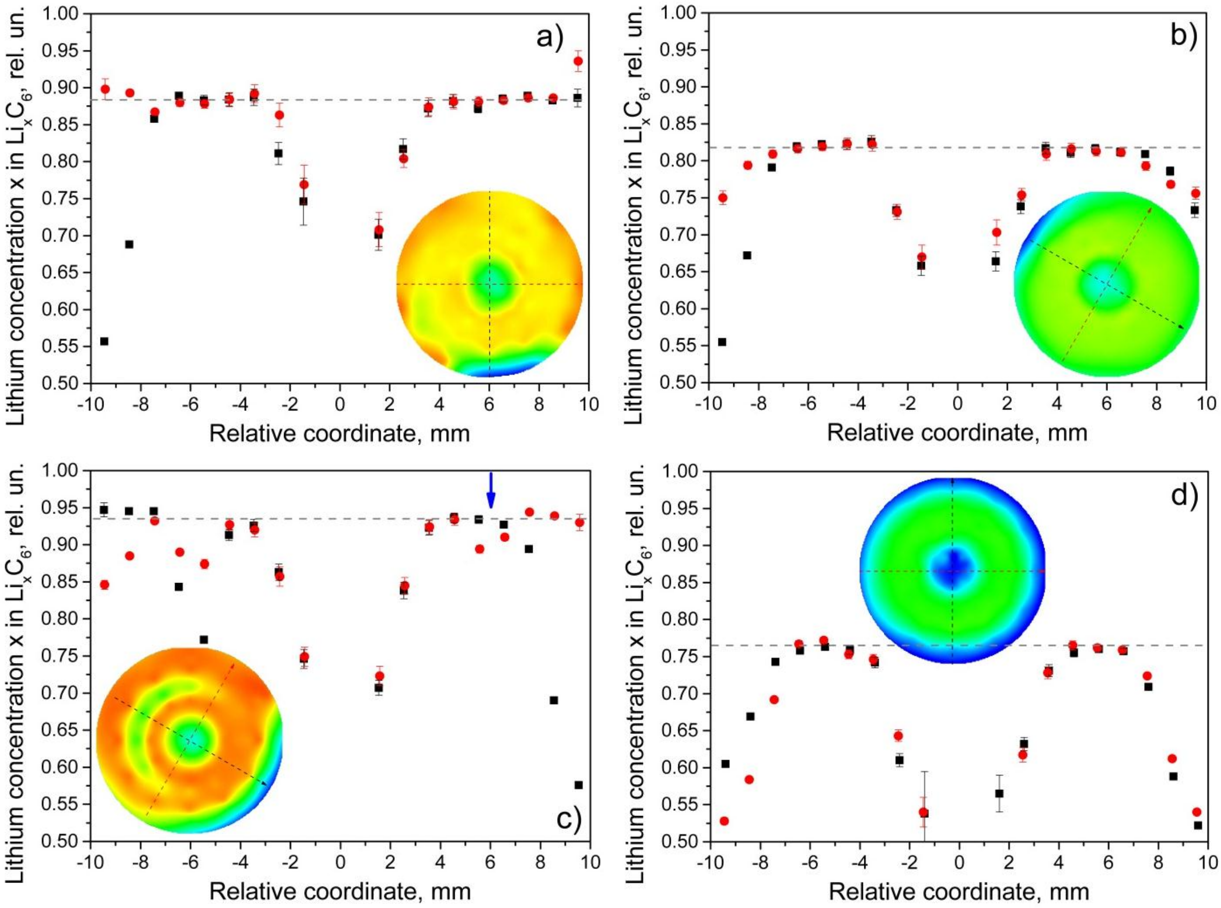

Fig. S6. Lithium concentration profiles along two chosen orthogonal directions (shown by black and red points, respectively) in cell 1 (a), cell 2 (b), cell 3 (c) and cell 4 (d). Plateau value x_p is shown by horizontal dashed line. Blue arrow indicates deviation from plateau in cell 3.

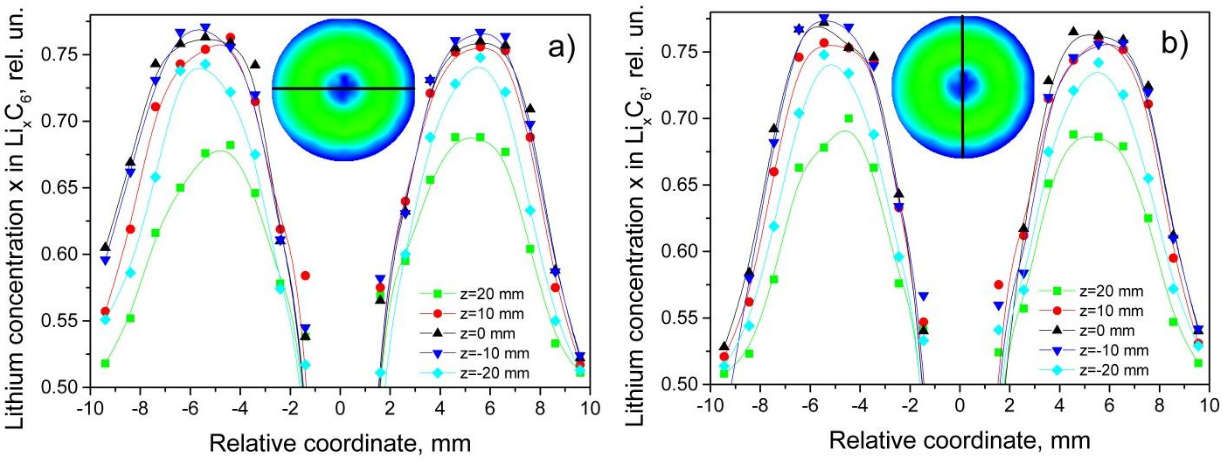

Fig. S7. Lithium concentration profiles along x and y axes in the anode of cell 4 collected at different heights z (corresponding to Fig. 5). Lines are shown as guides for the eyes.
